# Immunology of Tuberculosis

**DOI:** 10.4084/MJHID.2014.027

**Published:** 2014-04-07

**Authors:** Federica Bozzano, Francesco Marras, Andrea De Maria

**Affiliations:** 1 University of Genova, Italy; 2 Istituto G. Gaslini, Genova, Italy; 3 IRCCS AOU S. Martino-IST-Genova, Italy

## Abstract

MTB ranks as the first worldwide pathogen latently infecting one third of the population and the second leading cause of death from a single infectious agent, after the human immunodeficiency virus (HIV). The development of vigorous and apparently appropriate immune response upon infection with *M. tuberculosis* in humans and experimental animals conflict with failure to eradicate the pathogen itself and with its ability to undergo clinical latency from which it may exit. From a clinical standpoint, our views on MTB infection may take advantage from updating the overall perspective, that has quite changed over the last decade, following remarkable advances in our understanding of the manipulation of the immune system by M. tuberculosis and of the role of innate components of the immune response, including macrophages, neutrophils, dendritic cells and NK cells in the initial spread of MTB and its exit from latency. Scope of this review is to highlight the major mechanisms of MTB escape from immune control and to provide a supplementary translational perspective for the interpretation of innate immune mechanisms with particular impact on clinical aspects.

## Introduction

*Mycobacterium tuberculosis* (MTB) may be regarded as the most successful intracellular bacterium worldwide, in view of its world prevalence and distribution. MTB ranks as the second leading cause of death from a single infectious agent, after the human immunodeficiency virus (HIV). Indeed, about 8.7 million incident cases of pulmonary tuberculosis (TB) (range, 8.3 million to 9 million), equivalent to 125 cases per 100,000 population, were registered globally in 2011.^1^ Despite availability of antituberculous drugs for the last 50 years Mth is responsible for 1.5 million deaths every year with about one third of the world population having been in contact and latently infected.^1^,^2^

Since its first characterization by Robert Koch at the end of the 19^th^ century,^3^ intense efforts have led to the characterization of the manifold interactions of M. tuberculosis with the immune system, to a renewed study of its metabolism for the identification of new specific pathways subject to inhibition by new drugs, to the discovery of the mechanisms it uses to divert host defences and to the understanding of the broad spectrum of soluble factors and cells involved in its control.

Despite relevant advances over the last 20 years in our understanding of the broad outlines of mechanisms contributing to protective immunity to M. tuberculosis, relevant scientific and clinical challenges remain. The development of vigorous and apparently appropriate immune response upon infection with M. tuberculosis in humans and experimental animals conflicts with failure to eradicate the pathogen itself and with its ability to undergo clinical latency from which it may exit causing the bulk of overt clinical tubercular disease in everyday clinical life. In particular, our incomplete understanding of mechanisms potentially allowing complete eradication of M. tuberculosis once infection has taken place, and of those failing during latency - thus leading to reactivation of M. tuberculosis only in a subset (10–15%) of latently infected subjects^4^ - represent major hurdles towards effective second-generation vaccines and targeted treatment of latency.

The classical view of immunity to M. tuberculosis mainly recognizes participation of macrophages and cells of the adaptive immune system (CD4+ and CD8+ T lymphocytes) in the control of mycobacteria. From a clinical standpoint, our views on MTB infection may take advantage from updating the overall perspective, that has quite changed over the last decade, following remarkable advances in our understanding of the manipulation of the immune system by M. tuberculosis and of the innate component of the immune response. Over recent years, it has indeed become clear, that, in addition to adaptive mechanisms, innate immune responses are recruited by and against M. tuberculosis according to the time-frame of response recognizing early and late events after MTB entry.

The purpose of the present review is not to provide a comprehensive review of TB immunology, nor to provide in depth focus on the mechanisms of M. tuberculosis virulence and pathogenicity which appear elsewhere^5^,^6^ rather, scope of this review is to highlight the different actors of the immune response against M. tuberculosis and the major mechanisms of MTB escape and to provide a supplementary translational perspective for the interpretation of innate immune mechanisms with particular impact on clinical aspects. Renewed frameworks of interpretation of results from human and animal research and from clinical observations will help the updating and understanding of M. tuberculosis immunopathogenesis and facilitate the design of new vaccines, drugs and prevention strategies.

## Clinical Correlates of the Immune Response to *M. tuberculosis.*

**Figure 1:**
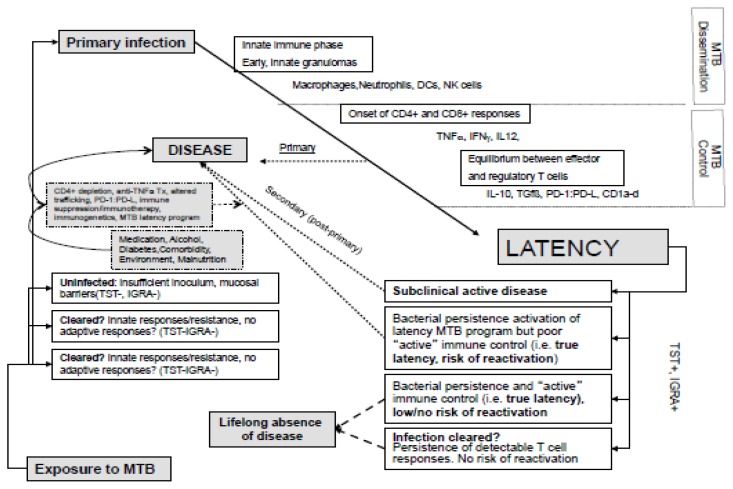
Diagram of the clinical courses and immune regulation accompanying exposure to and infection by *M. tuberculosis.*

Active Tuberculosis (TB) encompasses a range of clinical presentations and disease courses. Active TB occurs in two stages, either as the natural evolution of overwhelming *M. tuberculosis* replication following initial infection (Primary or primary-progressive TB), or resuming after a latent infection/containment of *M. tuberculosis* that may last many years following exposure (post-primary TB or reactivated TB). Both primary and post-primary TB occurs in only a minor fraction of those at risk, as a consequence of several factors that include both innate and adaptive immune responses. Primary TB is detected in up to 20% of those exposed to M. tuberculosis airborne inoculum, and post-primary TB with reactivation of M. tuberculosis from latency occurs at a rate of 0.1–0.5% per year with an estimated 5–10% lifetime risk of developing active TB.^4^,^7^ This heterogeneity of individual responses is associated to different immunogenotypic characteristics (extending from innate immune responses to adaptive immune control of *M. tuberculosis*) and is highlighted by the non-human primate model (*Cynomolgus macaque*, *Macaca fascicularis*) of experimental bronchial inoculation of a fixed MTB inoculum.^8^ Here, a whole spectrum of outcomes and pathological findings were observed, similar to what occurs after acute infection in humans. The outcomes of instillation of a defined inoculum was invariably infection, however the spectrum of pathology included macaques that progressed rapidly and succumbed to active disease, others that developed active disease over a more chronic course (including one who spontaneously resolved the infection), and those that displayed no evidence of disease even though they were clearly infected and had clinical characteristics similar to latent TB in humans. The heterogeneity of the host immune response extends beyond primary TB, and applies particularly to latent TB in humans -where only between 20–50% of latent close contacts of TB cases develop Tubercolin Skin Test (TST) reactions and 1–2% of these close contacts eventually develop active TB^9^,^10^ - and to latent TB in cynomolgus macaques.^8^,^11^ Thus, latent TB is reflective of a heterogeneous group of individuals:^12^ a) those who have *subclinical disease*, b)those who will progress to *primary active disease*; c) those who maintain *persistent, lifelong infection*; d)those who temporarily suppress infection but later develop active TB, possibly as a result of immunosuppression or some other event (i.e., *true latent infection*); e) those who are able -either through innate or adaptive immunity or the combination-to effectively clear the pathogen (Figure 1).

Unfortunately, no test is currently available to differentiate latent from active TB disease, as TST and interferon gamma-release assays (IGRA) simply report the presence of specific T cell responses irrespective of the clinical condition. Furthermore, there is no test to identify those latently infected individuals who may progress to active TB or those who have subclinical disease. Also in this case TST and IGRA do not help discriminate different clinical courses.

The ability to identify those individuals with latent TB who are at risk of reactivation would help target preventative therapy and devise individualized target treatment thereby increasing adherence and minimizing toxicity and costs. Using transcriptional profiling of leukocytes in whole peripheral blood by microarray analysis, a characteristic neutrophil-driven IFN-inducible 393 transcript-signature has been identified in patients with active TB.^13^ This transcript signature disappears by 2 months of effective treatment and correlates with the extent of radiographic involvement. Interestingly, 10–20% of patients with TB latency have a transcript signature similar to those with active disease. Although not proven yet, these patients might represent the minority of latent TB who will eventually progress to active TB years later. Leukocyte or purified cell population transcriptional analysis - also in other areas of chronic infections including HCV^14^ - is likely to become a useful future tool to identify the subset of patients with true latency who will develop post-primary reactivation and for whom chemoprophylaxis - or rather treatment - may be mandatory. The precise labelling of patients with different types of latency using molecular or immunological tools represents one of the main future challenges to individualize treatment of smoldering infection, prophylaxis of true latency and avoiding unnecessary toxicity for eradicated infection.

## Timing of Immune Responses and Granuloma Formation

Following the establishment of *M. tuberculosis* infection in the airways and lung parenchyma, the bacilli are believed to be phagocytosed by the alveolar macrophages^15^ and are taken up by neutrophils^16^ and dendritic cells (DCs).^17^ Over time, cells progressively assemble in a compact, organized aggregate of mature macrophages surrounded by fibroblasts and interspersed with neutrophils, DCs, Natural Killer cells, B cells, CD4+ and CD8+ T cells. This structure is a granuloma and has been historically and until recently considered to represent a concentrated effort of the immune system to sequester, wall off and eradicate M*. tuberculosis*.^18^,^19^ Recent evidences have however subverted the classic view that the granuloma is a host-protective structure. Indeed, different stages of the immune response to *M. tuberculosis* can be recognized, and granulomas are dynamic structures that are initially exploited by the bacterium to subvert the immune response, replicate and spread at other locations.

### Innate immune phase-granuloma dynamics

Upon MTB entry in the airways innate immune responses predominate. Early granulomas are composed of inflammatory macrophages, neutrophils and DCs that progressively accumulate upon recruitment. All the cells of the early granulomas engulf the mycobacteria and become infected. Pathogenic mycobacteria such as *M. tubercuolosis* and *M. marinum* in the zebrafish model have evolved multiple mechanisms to manipulate this cellular niche to their own advantage. Trafficking and maturation of phagosomes in which pathogenic mycobacteria reside is manipulated to prevent lysosomal killing and degradation.^20^ Surprisingly, in spite of overwhelming infection, macrophages and DCs in the early granuloma are inefficient in presenting *M. tuberculosis* antigens in the early granuloma to CD4+ T-cells.^21^ Efficient MTB Antigen (Ag)-presentation only takes place later in the lymphnode.^22^ Mycobacteria, as exemplified by the zebrafish-*M. marinum* model, exploit granuloma formation for their proliferation and dissemination in the infected host. Dynamic imaging studies reveal that macrophage move rapidly within granulomas, at speeds comparable to lymphocytes in a chemokine gradient.^23^ Movement is dictated by the RD1 virulence locus that is responsible for *M. tuberculosis* ESX-1 secretion system (which is lost in attenuated Bacillus Calmette-Guerin),^24^,^25^ and ceases when macrophages contact dying infected cells, thereby increasing the number of infected cells. Also the necrotic core of granuloma, which was regarded as being not involved in immune interactions, is crossed by infected and non-infected macrophages.^21^,^26^ Finally, tertiary lymphoid structures are found in granulomas in the lungs of mice.^27^ Here, macrophage and T-cell movement resemble those of T and B cell trafficking in secondary lymphoid organs,^21^,^28^ and chemokines are produced (CCL19, CCL21), which are characteristic chemoattractants for CCR7-bearing lymphocytes homing to lymphoid structures.^29^ Thus, despite macrophage, T-cell and DC entry into the granuloma in the early phase, these cells do not leave, and at the same time cannnot proceed to antigen presentation neither locally nor in lymphnodes.

### TNF-α vs. MMP-9 in granuloma formation

During early granuloma formation, TNFα has been historically considered instrumental to granuloma formation and to increase the ability of macrophage control of intracellular mycobacteria.^30^ This view is however challenged by the observation that in non-human primates TNFα–blockade results in disseminated disease with normal granuloma structure,^31^ and by similar findings in patients treated with anti-TNFα treatment.^32^,^33^ Indeed in the zebrafish model, TNFα increases pathogenic *M. marinum* death and its absence is associated with accelerated and increases granuloma formation.^34^ Rather than TNFα, the mechanism(s) underlying granuloma formation have been shown to involve induction of host matrix-metalloprotase-9 (MMP-9) production by macrophages and epithelial cells upon interaction with RD-1 locus-encoded, secreted ESAT-6.^25^,^35^ In line with this view, MMP-9 knockout mice have decreased granuloma formation and improved control of infection and has been found to be enriched in tissues and pleural fluid in human pulmonary TB.^36^

### Death matters: Apoptosis vs. Necrosis

During early phases, mycobacterial load is rapidly rising through granuloma formation with influx and infection of neutrophils and macrophages and cell death. While necrosis, with cell lysis, propagates locally viable mycobacteria and increases pathogen load, programmed cell-death or apoptosis maintains intact cellular membranes favoring cellular compartmentalization and mycobacterial containment.^37^,^38^ The type of cell death that is induced depends on the regulation of the lipid mediator eicosanoids prostaglandin E2 (PGE2, proapoptotic) and lipoxin A4 (LXA4, pronecrotic).^39^ Differences in eicosanoid pathway activity and regulation may contribute to inter individual differences in cross-presentation of *M. tuberculosis* by Dendritic Cells (DC), thus affecting also adaptive immune differences and the clinical evolution from infection to primary disease or to true latency.^40^ Neutrophils support *M. tuberculosis* replication and spread^16^,^41^ and may have dual roles in the early defense against the pathogen. Activation of antigen-specific CD4+ T cells is facilitated by neutrophils,^42^ however inhibition of neutrophil apoptosis by MTB determines their delayed activation.^43^

Therefore early - and sometimes late - granuloma formation does not “wall off” mycobacteria. The view of a mechanical containment in granulomas following TNFα induction is being replaced with a new perspective indicating that granuloma formation is induced by pathogenic mycobacteria through mechanism(s) including ESAT-6-induced^25^ MMP-9 production.^35^ Thus early granulomas favor increased macrophage accumulation, mycobacterial replication, and systemic MTB spread. Even adoptive transfer of Ag-specific CD4+ T cells shows that in this phase of the infection Mycobacteria are secluded in a protected niche within the granuloma^44^ and that intervention should target innate immune events (including anti-MMP-9^45^ or pro-apoptotic treatments), that predominate during the early phase but that persist also in later equilibrium phases of the infection.

Overall, therefore, the early stages of antimycobacterial immune responses are dominated by innate immune responses that have little immediate antimycobacterial effect and rather favor its spread and replication. The subsequent adaptive phase however builds on initial innate responses, which are eventually needed for antigen presentation and editing of adaptive responses.

## Adaptive Responses and Immune Equilibrium

The relatively small proportion of patients that progress to primary TB following infection by *M. tuberculosis* and of those that upon acquiring latent infection progress to post-primary disease should be regarded as a success of host defenses, even if latency consists in arrest of bacterial growth, not in bacterial sterilization.

The prominent characteristic of specific antimycobacterial adaptive responses is the long delay in onset and the need for their continuous persistence and effort to maintain latency. Adaptive responses are relevant to containment and control of MTB replication, involve IFN-γ-producing or poly-functional (IL-2, IFN-γ and TNFα) CD4+ and CD8+ T lymphocytes. Adaptive responses are delayed in the early granuloma, and ultimately rely on the presentation of specific mycobacterial antigens by DCs, under editing, control and help by NKT and NK cells.^46^,^47^ Initiation of the adaptive response begins in lymphnodes, where infected DC traffic after initial delay and persistence in peripheral tissues (alveoli and lung tissue) where even 100-fold higher bacterial concentrations are found.^22^,^48^ Further local delay in lung adaptive responses to *M. tuberculosis* is due to the influx of pathogen-specific CD4+ regulatory T cells generated in lymphnodes ad migrating to the tissue^49^, and by the direct inhibition of apoptosis by M. tuberculosis in infected neutrophils.^43^ Regulatory CD4+ T cells (Treg) are generated in lymphnodes together with Th1 (T helper 1) CD4+ T cells in the early phase of adaptive responses, and are responsible for failure to eradicate M. tuberculosis in the long run, as shown by adoptive transfer in the mice model^50^. In addition to the presence of Treg CD4+ T cells, also the expression of PD-1 on Ag-specific CD4+ T cells is a factor favoring M. tuberculosis persistence and survival once latency has been established in the mice model.^51^ Overall, however, survival to *M. tuberculosis* relies on the presence of CD4+ T cells which play a fundamental role in inhibiting its replication and protect from active disease. Indeed CD4 lymphopenic patients with or without HIV infection are at increased risk of developing active TB.^52^ Although CD4+ T lymphocytes have been considered to be the primary source of IFN-γ and to be protective through its secretion, this is not the case. CD4+ T cell depletion induces disease in mice while leaving unchanged lung tissue levels of IFN-γ.^53^ and conversely, IFN-γ deficiency still allows protection^54^. In mice and human models it appears that CD4+ T cells per se, rather than their production of IFN-γ may be protective. High IFN-γ levels in lung tissue and granuloma may be attributed to Ag-specific CD8+ T cells which produce IFN-γ and TNF-α and are involved in the control of *M. tuberculosis*,^55^ and to NK cells, that are the main IFN-γ producers involved in DC maturation and editing.^56^ HLA-E-dependent presentation of peptides to human CD8+ T cells may be also involved in the control of *M. tuberculosis* and has been found to comprise a dominant immune response in latently infected patients.^57^ Additional support for CD8+ T cell involvement in the control of *M. tuberculosis* is provided by the mycobacteriostatic effect of granulysin in CD8+ CTL granules^58^,^59^ and by disseminated infection in mice lacking HLA class I presentation pathways (e.g.ß2-microglobulin, transporter associated with antigen processing, TAP).^60^–^62^

## TB Reactivation

One of the most obscure areas in our understanding of the relationship of the immune system with *M. tuberculosis* is represented by the clinical transition from latency to post-primary TB. Factors underlying this transition are so far only partially understood, and there is no understanding of the precise mechanism(s) that induce transition to reactivation from a dormant state. Knowledge of these mechanism(s) would be of crucial importance, since this would allow i) identification and prophylactic/therapeutic targeting of the minority of patients with latent TB that will actually progress to reactivation, thus avoiding unnecessary potentially toxic and costly courses of chemoprophylaxis administered to those who would never need it in a lifetime; ii) monitoring and prediction of the exact moment when *M. tuberculosis* would exit latency in these patients and iii) devise immune intervention/vaccination strategies to boost immune control of M. tuberculosis of this selected minority of patients.

Although it has been held for a long time that the merit for latency persistance should be attributed to the immune system, evidences accumulated in the last years point out that also *M. tuberculosis* actively participates to this process. *M. tuberculosis* activates a bacterial regulon controlled by the DosR-DosS signal transduction system in the presence of local hypoxia, carbon monoxide or nitric oxide.^63^,^64^ In the presence of these stimuli, which are believed to be prevalent during latency, *M. tuberculosis* activates the expression of a set of genes allowing the use of alternative energy sources. Within this program, it expresses genes whose products are recognized by T cells. The regulation of this set of genes during latency and the shut off of the transcriptional program that is active during the replicative active phase of *M. tuberculosis* life cycle implies that specific mycobacterial epitopes become available during latency while others may disappear and no longer be recognized.^65^,^66^

*M. tuberculosis* encodes two additional gene clusters that are involved in exit from latency, whose regulation contributes to determine the outcome of infection. Five encoded proteins resemble the “resuscitation-promoting factor (Rpf)” produced by *M. luteus* to recover from nutrient-starved latent phase.^67^ Deletion of Rpf-like genes of *M. tuberculosis* impairs mycobacterium recovery from latency^68^,^69^ in a mice model. Interestingly, double RpfAB knockouts have a different interaction with innate immune mechanisms and induce higher amounts of TNFα and IL-6 in infected macrophages. Rpf-like gene products therefore provide a clockwork for exit from latency and also a system to modulate innate responses thus favouring mycobacterial growth. Finally, an 88 toxin-antitoxin gene pair system is also encoded by *M. tuberculosis* and its transcription is involved in the decision to maintain latency or progress to overt replication and virulence.^70^

In view of the above data, shedding further light on the fine-tuning mechanisms employed by M. tuberculosis to regulate its access to and exit from latency represents a crucial step with relevant clinical and immune bearing. Immunoprophylactic prevention of exit from latency may, for example, require different antigen and epitope-targeting compared to those encoded by pathogenic replicating bacteria during primary invasion. Also, targeting of some of these transcripts/proteins may provide new tools for antibacterial treatment of early reactivation.

In general, exit from latency into post-primary TB is regarded as a so far poorly characterized consequence of “immune weakening”, and represents and event that is not predictable according the when, who, and where questions. As mentioned above, among any cohort of latently infected subjects it is impossible to predict who will fall in the 10% that eventually will experience reactivation, when this will take place or where the escape from immune control and exit from *M. tuberculosis* latency program will eventually occur (although this will occur in the lungs in 85% of the cases due to the high mycobacterial burden in this site).

Regardless of bacterial virulence factors involved in latency exit, two specific well characterized mechanisms are known to increase the likelihood of reactivation. The first involves quantitative and qualitative depletion of CD4+ T cells, while the other is represented by impairment of TNFα signaling. Immune deficiencies leading to CD4+ T cell loss, including HIV-1 infection, are associated to increased risk of *M. tuberculosis* reactivation^71^. The risk of TB reactivation during HIV is associated not only to quantitative defects of CD4+ T-cell counts, since many patients develop TB and AIDS well before CD4+ T-cell counts decrease below 350-200/μl. Selective targeting of TB-specific CD4+ T-cells, functional derangement of CD4+ T cells with skewing of polyfunctionality (IFN-γ, TNFα and IL-2 production) or skewing of cytokine production patterns have been advocated.^72^,^73^,^74^–^77^ No precise CD4-associated mechanism has been so far pinpointed, however, to explain how CD4+ T cells accomplish control of *M. tuberculosis* in some patients but fail in others. With regard to TNFα, it has been well established that neutralization of TNFα, particularly in the context of monoclonal antibody treatment,^78^,^79^ dramatically increases the chances of TB reactivation. In vitro, TNFα production and signaling in monocytes controls *M. tuberculosis* replication,^34^ as also shown with comparative use of RpfAB knockouts and wildtype strains in vitro.^68^

Additional mechanisms have been suggested to be involved in the exit from latency, such as programmed death receptors and ligands (PD-1/PD-L1). Increased blood expression of PD-L1 occurs during active TB and is predominantly due to its expression by neutrophils.^80^ In addition, PD-1 expression is associated with different functional profiles in CD8+ antigen specific CTLs in active and latent TB^81^, thus suggesting different functional predominance in the two conditions, and the crosstalk between innate and adaptive elements of the immune response. The ability to modulate gene expression and to shift antigenic expression during overt replication and latency may represent one of the mechanisms of evasion from the control by the host immune responses. The expression of different gene products during specific and different phases of the disease course may represent a mechanism for mycobacterial evasion from CD4- and CD8-specific T cell responses. For example, ESAT-6 and Ag85B represent major antigenic targets for CD4+ and CD8+ T cells and are actively expressed during overt infection. However, *M. tuberculosis* downregulates their expression as soon as specific CD4+ T cells appear in the mouse model, thus favoring the persistence of the pathogen.^82^,^83^

Altogether the increased frequency of TB reactivation in CD4 depleted patients (e.g. HIV--blocking treatments provide evidence that these represent two major elements contributing to persistent and successful control of *M. tuberculosis* replication and should be regarded as a correlate of efficient pathogen control. Since they represent respectively adaptive and innate arms of host anti-infective defenses, it is tempting to consider that exit from latency into overt disease may be due to modulation of either arm, and possibly through as yet poorly acknowledged pathways.

## Involvement of NK Cells in Early and Late Events Affecting Individual Disease Course

The attention on early innate events leading to permissive granuloma formation, and the subsequent development of a highly effective control of *M. tuberculosis* has so far concentrated on crucial events in monocytes, macrophages, neutrophils and dendritic cells. NK cell involvement has been left out of focus despite accumulating evidences of their involvement in the path of innate mechanisms leading to CD8+ and CD4+ adaptive responses. Several lines of evidence point to NK cell involvement at several steps along the path of control - or lack thereof - of *M. tuberculosis* replication and spread during primary seeding, in the control/exit from latency, and contributing to the immune response to vaccination and to innate resistance to infection. This paragraph is aimed at putting these aspects in frame.

Not only CD4+ and CD8+ T cells, but also NK cells have been shown to play a crucial role in killing of *M. tuberculosis* in human monocytes through production of IFN-γ.^84^ In the RAG(−/−)T-cell deficient mouse model *M. tuberculosis* stimulates NK-cell dependent IFN-γ production in naive spleen and lung cells, and NK cell-knockout or anti-IFN-γ-treated animals display dramatically increased susceptibility.^85^

NK cell may interact specifically with both infected macrophages and directly with mycobacteria through multiple receptors, thus anticipating a direct NK cell involvement in the recognition of mycobacteria upon entry in the lung tissue and throughout later events contributing to the generation of adaptive responses. The most physiologically crucial direct interplay of mycobacteria with NK cells is represented by the interaction with TLR2 (Toll-Like Receptor 2)^86^ possibly via binding to peptidoglycan.^87^ A direct contact of mycobacterial mycolic acids and arabinogalactan with NK cell-triggering natural cytotoxicity receptor NKp44 has also been suggested.^87^,^88^ More importantly, mycobacteria-infected macrophages are directly recognized and lysed by NK cells via NKG2D and NKp46 that recognize ULBP1 and vimentin whose expression on macrophages is upregulated upon infection.^89^–^91^ BCG-exposed macrophages (M0 and M2) in addition induce strong activation of resting NK cells in vitro leading to their production of IFN-γ and to cytotoxic activity induction.^92^ Overall, IFN-γ is produced not only by Ag-specific CD4+ T cells during late events accompanying establishment of adaptive immune responses, but also by NK cells –together with TNF-α - throughout early and later events after mycobacterial entry and spread^93^,^94^ and also maturing NK cells may contribute to BCG-induced immune responses with IFN-γ production.^95^

NK cells positively modulate adaptive immune responses against mycobacteria, and thus contribute to the mechanisms that ultimately lead to control of *M. tuberculosis* replication through influx of antigen-specific CD4+ and CD8+ T cells in areas of *M. tuberculosis* replication in macrophages. This is accomplished via induction of maturation of immature dendritic cells (iDC), reciprocal activation of NK cells by infected iDC with selection of optimally mature DC by NK cells.^46^,^56^ In addition, NK cells interacting through CD40L with Ag-specific CD8+ CTL also contribute to CD8+ CTL killing of infected macrophages and to their IFN-γ production^96^ and to direct control of *M. tuberculosis* growth.^97^ Finally, lysis of FoxP3+CD4+ Treg by NK cells^98^ provides an additional role played by these cells in the control of mycobacterial replication and spread thus dynamically counteracting the influx of CD4+ Treg cells in the granuloma that prevent early clearance of *M. tuberculosis*.^50^

Negative regulatory mechanisms for NK cell function with regard to *M. tuberculosis* infection have been described by the possibility to express de novo PD-1 and PD-L, thus dampening their activity.^99^ In addition, induction of CD1d on mycobacteria-infected monocytes negatively modulates NK cell triggering and induction of monocyte apoptosis and *M. tuberculosis* killing through interaction with inhibitory receptors expressed on NK cells.^100^,^101^ Finally, escape from NK cell-induced killing/apoptosis and of mycobacterial killing may occur in M2 monocytes.^92^

The spectrum of different mechanisms that may cause NK cell intervention during *M. tuberculosis* invasion and possibly during latency, and the array of mechanisms that negatively balance NK cell anti-mycobacterial function suggest that interindividual differences in the regulation of NK cells may contribute, in addition to other innate and adaptive variability, to the wide differences in clinical courses commonly observed after acute infection and in exit from latency in humans and in non-human primate models.^8^ In addition to be recruited to and detected in lung tissue granulomas of TB patients, NK cells show dramatic interindividual differences in IFNγ and TNFα production in healthy donors with up to 1000-fold variation upon BCG or H37Rv challenge.^94^ Thus, modulation of NK cell function, either inherent or acquired through exogenous factors, may underlie differences in clinical courses and thus help explain and possibly predict the disease course. While the majority of latently infected individuals will never experience reactivation, a fraction of patients develops re-activation through as yet poorly understood mechanisms.^12^ In this regard, regulation of NK cell function and receptor expression modulation could contribute to determine exit from latency. Indeed, patients with TB at reactivation have dramatically decreased expression of NKp30 and NKp46 natural cytotoxicity receptors.^102^ These receptors are involved respectively in DC recognition/DC editing with downstream shaping of adaptive responses and in the recognition of infected macrophages.^46^,^47^,^90^,^92^,^103^ Accordingly, decreased NCR (NKp46, NKp30) expression at reactivation is accompanied by relevant decreases in NK cell cytotoxicity and defective IFNγ production upon activation in vitro.^102^ With specific treatment, TB patients fully recovering clinically display a transcriptional signature of improvement in their PBMC,^13^ recover NK cell IFNγ production, but do not recover NKp30 and NKp46 expression.^102^

Specific functional skewing of other cells or molecules of the innate immune system are likely to play significant roles in this context. The recent finding that TLR-2 and TLR-9 polymorphisms are associated to an increased risk of TB in different populations^104^,^105^ possibly due to attenuation of receptor signalling^89^,^106^ coupled with the expression and relevance of these two TLRs also for NK cell activation^86^,^107^,^108^ suggests that multiple non-mutually exclusive mechanisms may contribute the events that lead to exit from latency in infected individuals. Therefore, both acquired environmental factors as well as inherent HLA-unrelated (e.g.:triggering receptor expression-NCR modulation) and HLA-related (e.g. inhibitory receptor- KIR-carriage/expression) mechanisms are likely to influence NK cell function and their contribution to the decreased innate immune surveillance during exit from latency.

## Conclusion

An increasingly focused picture of the early events leading to disease progression or establishment of latency has been provided in recent years by the investigation of innate immune mechanism(s) involving macrophages, neutrophils, DCs and NK cells, by the development of advanced animal models and by translational research in human disease addressing key questions on immune correlates of *M. tuberculosis* infection.

All the components of innate immune responses provide relevant contributions to the control of *M. tuberculosis* by antigen-specific adaptive T cell responses. The knowledge of these mechanism(s) will allow future development of vaccination strategies, monitoring of vaccine efficacy in selected individuals with specific innate immune response patterns, and in the identification of the minority of latently infected individuals who will develop reactivation post-primary disease.

## References

[1] Who (2012). Global tuberculosis report 2012.

[2] Dye C, Scheele S, Dolin P, Pathania V, Raviglione MC, for the WHOGSaMP (1999). Global burden of tuberculosis: Estimated incidence, prevalence, and mortality by country. JAMA.

[3] Koch R (1882). The etiology of tuberculosis. Berl Klin Wochenschr.

[4] Vynnycky E, Fine PEM (2000). Lifetime Risks, Incubation Period, and Serial Interval of Tuberculosis. American Journal of Epidemiology.

[5] Cooper AM (2009). Cell-Mediated Immune Responses in Tuberculosis. Annual Review of Immunology.

[6] Philips JA, Ernst JD (2012). Tuberculosis Pathogenesis and Immunity. Annual Review of Pathology: Mechanisms of Disease.

[7] Dye C, Scheele S, Dolin P (1999). Global burden of tuberculosis: Estimated incidence, prevalence, and mortality by country. JAMA.

[8] Lin PL, Rodgers M, Smith Lk (2009). Quantitative Comparison of Active and Latent Tuberculosis in the Cynomolgus Macaque Model. Infection and Immunity.

[9] Marks SM, Taylor Z, Qualls NL, Shrestha-Kuwahara RJ, Wilce MA, Nguyen CH (2000). Outcomes of Contact Investigations of Infectious Tuberculosis Patients. American Journal of Respiratory and Critical Care Medicine.

[10] Jereb JES, Joglar OT, Moore M, Taylor Z (2003). Tuberculosis contact investigations: outcomes in selected areas of the United States, 1999. Int J Tuberc Lung Dis.

[11] Capuano SV, Croix DA, Pawar S (2003). Experimental Mycobacterium tuberculosis Infection of Cynomolgus Macaques Closely Resembles the Various Manifestations of Human M. tuberculosis Infection. Infection and Immunity.

[12] Barry CE, Boshoff HI, Dartois V (2009). The spectrum of latent tuberculosis: rethinking the biology and intervention strategies. Nat Rev Micro.

[13] Berry MPR, Graham CM, McNab FW (2010). An interferon-inducible neutrophil-driven blood transcriptional signature in human tuberculosis. Nature.

[14] Ascierto M, Alter H, Bozzano F (2013). Genomic scale analysis of NK cells impact on response to IFN-alpha. Journal for ImmunoTherapy of Cancer.

[15] Schlesinger L (1996). Entry of Mycobacterium tuberculosis into mononuclear phagocytes. Curr Top Microbiol Immunol.

[16] Eum S-Y, Kong J-H, Hong M-S (2010). NEutrophils are the predominant infected phagocytic cells in the airways of patients with active pulmonary tb. CHEST Journal.

[17] Wolf AJ, Linas B, Trevejo-Nu-ez GJ (2007). Mycobacterium tuberculosis Infects Dendritic Cells with High Frequency and Impairs Their Function In Vivo. The Journal of Immunology.

[18] Longo DLFA, Kasper DL, Hauser SL, Jameson JL, Loscalzo J (2012). Harrison’s Principles of Internal Medicine.

[19] Mandell GL, Bennett JE, Dolin R (2010). Mandell, Douglas, and Bennett’s Principles and Practice of Infectious Diseases.

[20] Sturgill-Koszycki SSP, Chakraborty P, Haddix PL, Collins HL, Fok AKAR, Gluck SL, Heuser J, Russell DG (1994). Lack of acidification in Mycobacterium phagosomes produced by exclusion of the vesicular proton-ATPase. Science.

[21] Egen Jackson G, Rothfuchs Antonio G, Feng Carl G, Horwitz Marcus A, Sher A, Germain Ronald N (2011). Intravital Imaging Reveals Limited Antigen Presentation and T Cell Effector Function in Mycobacterial Granulomas. Immunity.

[22] Wolf AJ, Desvignes L, Linas B (2008). Initiation of the adaptive immune response to Mycobacterium tuberculosis depends on antigen production in the local lymph node, not the lungs. The Journal of Experimental Medicine.

[23] Davis JM, Ramakrishnan L (2009). The Role of the Granuloma in Expansion and Dissemination of Early Tuberculous Infection. Cell.

[24] Volkman HE, Clay H, Beery D, Chang JCW, Sherman DR, Ramakrishnan L (2004). Tuberculous Granuloma Formation Is Enhanced by a Mycobacterium Virulence Determinant. PLoS Biol.

[25] Volkman HE, Pozos TC, Zheng J, Davis JM, Rawls JF, Ramakrishnan L (2010). Tuberculous Granuloma Induction via Interaction of a Bacterial Secreted Protein with Host Epithelium. Science.

[26] Cosma CL, Humbert O, Ramakrishnan L (2004). Superinfecting mycobacteria home to established tuberculous granulomas. Nat Immunol.

[27] Ulrichs T, Kosmiadi GA, Trusov V (2004). Human tuberculous granulomas induce peripheral lymphoid follicle-like structures to orchestrate local host defence in the lung. The Journal of Pathology.

[28] Stoll S, Delon J, Brotz TM, Germain RN (2002). Dynamic Imaging of T Cell-Dendritic Cell Interactions in Lymph Nodes. Science.

[29] Kahnert A, Höpken UE, Stein M, Bandermann S, Lipp M, Kaufmann SHE (2007). Mycobacterium tuberculosis Triggers Formation of Lymphoid Structure in Murine Lungs. Journal of Infectious Diseases.

[30] Bean AGD, Roach DR, Briscoe H (1999). Structural Deficiencies in Granuloma Formation in TNF Gene-Targeted Mice Underlie the Heightened Susceptibility to Aerosol Mycobacterium tuberculosis Infection, Which Is Not Compensated for by Lymphotoxin. The Journal of Immunology.

[31] Lin PL, Myers A, Smith LK (2010). Tumor necrosis factor neutralization results in disseminated disease in acute and latent Mycobacterium tuberculosis infection with normal granuloma structure in a cynomolgus macaque model. Arthritis & Rheumatism.

[32] Garcia Vidal C, Rodríguez Fernández S, Martínez Lacasa J (2005). Paradoxical Response to Antituberculous Therapy in Infliximab-Treated Patients with Disseminated Tuberculosis. Clinical Infectious Diseases.

[33] Iliopoulos A, Psathakis K, Aslanidis S, Skagias L, Sfikakis PP (2006). Tuberculosis and granuloma formation in patients receiving anti-TNF therapy [SHORT COMMUNICATION]. The International Journal of Tuberculosis and Lung Disease.

[34] Clay H, Volkman HE, Ramakrishnan L (2008). Tumor Necrosis Factor Signaling Mediates Resistance to Mycobacteria by Inhibiting Bacterial Growth and Macrophage Death. Immunity.

[35] Taylor JL, Hattle JM, Dreitz SA (2006). Role for Matrix Metalloproteinase 9 in Granuloma Formation during Pulmonary Mycobacterium tuberculosis Infection. Infection and Immunity.

[36] Sheen P, O’Kane CM, Chaudhary K (2009). High MMP-9 activity characterises pleural tuberculosis correlating with granuloma formation. European Respiratory Journal.

[37] Behar SM, Martin CJ, Booty MG (2011). Apoptosis is an innate defense function of macrophages against Mycobacterium tuberculosis. Mucosal Immunol.

[38] Hinchey J, Lee S, Jeon BY (2007). Enhanced priming of adaptive immunity by a proapoptotic mutant of Mycobacterium tuberculosis. The Journal of Clinical Investigation.

[39] Chen M, Divangahi M, Gan H (2008). Lipid mediators in innate immunity against tuberculosis: opposing roles of PGE2 and LXA4 in the induction of macrophage death. The Journal of Experimental Medicine.

[40] Divangahi M, Desjardins D, Nunes-Alves C, Remold HG, Behar SM (2010). Eicosanoid pathways regulate adaptive immunity to Mycobacterium tuberculosis. Nat Immunol.

[41] Abadie V, Badell E, Douillard P (2005). Neutrophils rapidly migrate via lymphatics after Mycobacterium bovis BCG intradermal vaccination and shuttle live bacilli to the draining lymph nodes. Blood.

[42] Blomgran R, Ernst JD (2011). Lung Neutrophils Facilitate Activation of Naive Antigen-Specific CD4+ T Cells during Mycobacterium tuberculosis Infection. The Journal of Immunology.

[43] Blomgran R, Desvignes L, Briken V, Ernst Joel D (2012). Mycobacterium tuberculosis Inhibits Neutrophil Apoptosis, Leading to Delayed Activation of Naive CD4 T cells. Cell Host & Microbe.

[44] Gallegos AM, Pamer EG, Glickman MS (2008). Delayed protection by ESAT-6–specific effector CD4+ T cells after airborne M. tuberculosis infection. The Journal of Experimental Medicine.

[45] Walker NF, Clark SO, Oni T (2012). Doxycycline and HIV Infection Suppress Tuberculosis-induced Matrix Metalloproteinases. American Journal of Respiratory and Critical Care Medicine.

[46] Vivier E, Raulet DH, Moretta A (2011). Innate or Adaptive Immunity? The Example of Natural Killer Cells. Science.

[47] Moretta A (2002). Natural killer cells and dendritic cells: rendezvous in abused tissues. Nat Rev Immunol.

[48] Gallegos AM, Pamer EG, Glickman MS (2008). Delayed protection by ESAT-6, Äìspecific effector CD4+ T cells after airborne M. tuberculosis infection. The Journal of Experimental Medicine.

[49] Shafiani S, Tucker-Heard GÄ, Kariyone A, Takatsu K, Urdahl KB (2010). Pathogen-specific regulatory T cells delay the arrival of effector T cells in the lung during early tuberculosis. The Journal of Experimental Medicine.

[50] Kursar M, Koch M, Mittrvºcker H-W (2007). Cutting Edge: Regulatory T Cells Prevent Efficient Clearance of Mycobacterium tuberculosis. The Journal of Immunology.

[51] Barber DL, Mayer-Barber KD, Feng CG, Sharpe AH, Sher A (2011). CD4 T Cells Promote Rather than Control Tuberculosis in the Absence of PD-1, ÄìMediated Inhibition. The Journal of Immunology.

[52] Geldmacher C, Schuetz A, Ngwenyama N (2008). Early Depletion of Mycobacterium tuberculosis-Specific T Helper 1 Cell Responses after HIV-1 Infection. Journal of Infectious Diseases.

[53] Scanga CA, Mohan VP, Yu K (2000). Depletion of Cd4+ T Cells Causes Reactivation of Murine Persistent Tuberculosis despite Continued Expression of Interferon = and Nitric Oxide Synthase 2. The Journal of Experimental Medicine.

[54] Cowley SnC, Elkins KL (2003). CD4+ T Cells Mediate IFN-g-Independent Control of Mycobacterium tuberculosis Infection Both In Vitro and In Vivo. The Journal of Immunology.

[55] Lalvani A, Brookes R, Wilkinson RJ (1998). Human cytolytic and interferon gamma-secreting CD8+ T lymphocytes specific for Mycobacterium, tuberculosis. Proceedings of the National Academy of Sciences.

[56] Ferlazzo G, Morandi B, D’Agostino A (2003). The interaction between NK cells and dendritic cells in bacterial infections results in rapid induction of NK cell activation and in the lysis of uninfected dendritic cells. European Journal of Immunology.

[57] Heinzel AS, Grotzke JE, Lines RA (2002). HLA-E-ìndependent Presentation of Mtb-derived Antigen to Human CD8+ T Cells. The Journal of Experimental Medicine.

[58] Stenger S, Hanson DA, Teitelbaum R (1998). An Antimicrobial Activity of Cytolytic T Cells Mediated by Granulysin. Science.

[59] Stegelmann F, Bastian M, Swoboda K (2005). Coordinate Expression of CC Chemokine Ligand 5, Granulysin, and Perforin in CD8+ T Cells Provides a Host Defense Mechanism against Mycobacterium tuberculosis. The Journal of Immunology.

[60] Mogues T, Goodrich ME, Ryan L, LaCourse R, North RJ (2001). The Relative Importance of T Cell Subsets in Immunity and Immunopathology of Airborne Mycobacterium tuberculosis Infection in Mice. The Journal of Experimental Medicine.

[61] Flynn JL, Goldstein MM, Triebold KJ, Koller B, Bloom BR (1992). Major histocompatibility complex class I-restricted T cells are required for resistance to Mycobacterium tuberculosis infection. Proceedings of the National Academy of Sciences.

[62] Behar SM, Dascher CC, Grusby MJ, Wang C-R, Brenner MB (1999). Susceptibility of Mice Deficient in CD1D or TAP1 to Infection with Mycobacterium tuberculosis. The Journal of Experimental Medicine.

[63] Park H-D, Guinn KM, Harrell MI (2003). Rv3133c/dosR is a transcription factor that mediates the hypoxic response of Mycobacterium tuberculosis. Molecular Microbiology.

[64] Sherman DR, Voskuil M, Schnappinger D, Liao R, Harrell MI, Schoolnik GK (2001). Regulation of the Mycobacterium tuberculosis hypoxic response gene encoding ±-crystallin. Proceedings of the National Academy of Sciences.

[65] Black GF, Thiel BA, Ota MO (2009). Immunogenicity of Novel DosR Regulon-Encoded Candidate Antigens of Mycobacterium tuberculosis in Three High-Burden Populations in Africa. Clinical and Vaccine Immunology.

[66] Govender L, Abel B, Hughes EJ (2010). Higher human CD4 T cell response to novel Mycobacterium tuberculosis latency associated antigens Rv2660 and Rv2659 in latent infection compared with tuberculosis disease. Vaccine.

[67] Chao MCRE (2010). Letting sleeping dos lie: does dormancy play a role in tuberculosis?. Annu Rev Microbiol.

[68] Russell-Goldman E, Xu J, Wang X, Chan J, Tufariello JM (2008). A Mycobacterium tuberculosis Rpf Double-Knockout Strain Exhibits Profound Defects in Reactivation from Chronic Tuberculosis and Innate Immunity Phenotypes. Infection and Immunity.

[69] Tufariello JM, Mi K, Xu J (2006). Deletion of the Mycobacterium tuberculosis Resuscitation-Promoting Factor Rv1009 Gene Results in Delayed Reactivation from Chronic Tuberculosis. Infection and Immunity.

[70] Ramage HR, Connolly LE, Cox JS (2009). Comprehensive Functional Analysis of Mycobacterium tuberculosisToxin-Antitoxin Systems: Implications for Pathogenesis, Stress Responses, and Evolution. PLoS Genet.

[71] Kwan CK, Ernst JD (2011). HIV and Tuberculosis: a Deadly Human Syndemic. Clinical Microbiology Reviews.

[72] Geldmacher C, Ngwenyama N, Schuetz A (2010). Preferential infection and depletion of Mycobacterium tuberculosis, Äìspecific CD4 T cells after HIV-1 infection. The Journal of Experimental Medicine.

[73] Day CL, Mkhwanazi N, Reddy S (2008). Detection of Polyfunctional Mycobacterium tuberculosis, ÄìSpecific T Cells and Association with Viral Load in HIV-1, ÄìInfected Persons. Journal of Infectious Diseases.

[74] Kalsdorf B, Scriba TJ, Wood K (2009). HIV-1 Infection Impairs the Bronchoalveolar T-Cell Response to Mycobacteria. American Journal of Respiratory and Critical Care Medicine.

[75] Caccamo N, Guggino G, Joosten SA (2010). Multifunctional CD4+ T cells correlate with active Mycobacterium tuberculosis infection. European Journal of Immunology.

[76] Marin ND, Paris SC, Rojas M, Garcia LF (2012). Functional profile of CD4+ and CD8+ T cells in latently infected individuals and patients with active TB. Tuberculosis (Edinburgh, Scotland).

[77] Perreau M, Rozot V, Welles HC (2013). Lack of Mycobacterium tuberculosis–specific interleukin-17A–producing CD4+ T cells in active disease. European Journal of Immunology.

[78] Wallis RS (2008). Tumour necrosis factor antagonists: structure, function, and tuberculosis risks. The Lancet Infectious Diseases.

[79] Harris J, Keane J (2010). How tumour necrosis factor blockers interfere with tuberculosis immunity. Clinical & Experimental Immunology.

[80] McNab FW, Berry MPR, Graham CM (2011). Programmed death ligand 1 is over-expressed by neutrophils in the blood of patients with active tuberculosis. European Journal of Immunology.

[81] Rozot V, Vigano S, Mazza-Stalder J (2013). Mycobacterium tuberculosis-specific CD8+ T cells are functionally and phenotypically different between latent infection and active disease. European Journal of Immunology.

[82] Shi L, North R, Gennaro ML (2004). Effect of Growth State on Transcription Levels of Genes Encoding Major Secreted Antigens of Mycobacterium tuberculosis in the Mouse Lung. Infection and Immunity.

[83] Bold TD, Banaei N, Wolf AJ, Ernst JD (2011). Suboptimal Activation of Antigen-Specific CD4+ Effector Cells Enables Persistence of M. tuberculosis In Vivo. PLoS Pathog.

[84] Yoneda T, Ellner JÄ (1998). CD4+ T Cell and Natural Killer Cell-dependent Killing of Mycobacterium tuberculosis by Human Monocytes. American Journal of Respiratory and Critical Care Medicine.

[85] Feng CG, Kaviratne M, Rothfuchs AG (2006). NK Cell-Derived IFN- = Differentially Regulates Innate Resistance and Neutrophil Response in T Cell-Deficient Hosts Infected with Mycobacterium tuberculosis. The Journal of Immunology.

[86] Marcenaro E, Ferranti B, Falco M, Moretta L, Moretta A (2008). Human NK cells directly recognize Mycobacterium bovis via TLR2 and acquire the ability to kill monocyte-derived DC. International Immunology.

[87] Esin S, Counoupas C, Aulicino A (2013). Interaction of Mycobacterium tuberculosis Cell Wall Components with the Human Natural Killer Cell Receptors NKp44 and Toll-Like Receptor 2. Scandinavian Journal of Immunology.

[88] Esin S, Batoni G, Counoupas C (2008). Direct Binding of Human NK Cell Natural Cytotoxicity Receptor NKp44 to the Surfaces of Mycobacteria and Other Bacteria. Infection and Immunity.

[89] Vankayalapati R, Wizel B, Weis SE (2002). The NKp46 receptor contributes to NK cell lysis of mononuclear phagocytes infected with an intracellular bacterium. J Immunol.

[90] Vankayalapati R, Garg A, Porgador A (2005). Role of NK Cell-Activating Receptors and Their Ligands in the Lysis of Mononuclear Phagocytes Infected with an Intracellular Bacterium. The Journal of Immunology.

[91] Garg A, Barnes PF, Porgador A (2006). Vimentin Expressed on Mycobacterium tuberculosis-Infected Human Monocytes Is Involved in Binding to the NKp46 Receptor. The Journal of Immunology.

[92] Bellora F, Castriconi R, Dondero A (2011). The interaction of human natural killer cells with either unpolarized or polarized macrophages results in different functional outcomes. Proceedings of the National Academy of Sciences.

[93] Smith S, Lalor M, Gorak-Stolinska P (2010). Mycobacterium tuberculosis PPD-induced immune biomarkers measurable in vitro following BCG vaccination of UK adolescents by multiplex bead array and intracellular cytokine staining. BMC Immunology.

[94] Portevin D, Via LE, Eum S, Young D (2012). Natural killer cells are recruited during pulmonary tuberculosis and their ex vivo responses to mycobacteria vary between healthy human donors in association with KIR haplotype. Cellular Microbiology.

[95] Marras F, Bozzano F, Bentivoglio G (2012). Receptor modulation and functional activation of human CD34+Lin--derived immature NK cells in vitro by Mycobacterium bovis Bacillus Calmette-Guerin (BCG). European Journal of Immunology.

[96] Vankayalapati R, Klucar P, Wizel B (2004). NK cells regulate CD8+ T cell effector function in response to an intracellular pathogen. J Immunol.

[97] Guerra C, Johal K, Morris D (2012). Control of Mycobacterium tuberculosis growth by activated natural killer cells. Clinical & Experimental Immunology.

[98] Dhiman R, Periasamy S, Barnes PF (2012). NK1.1+ Cells and IL-22 Regulate Vaccine-Induced Protective Immunity against Challenge with Mycobacterium tuberculosis. The Journal of Immunology.

[99] Alvarez IB, Pasquinelli V, Jurado JO (2010). Role Played by the Programmed Death-1-Programmed Death Ligand Pathway during Innate Immunity against Mycobacterium tuberculosis. Journal of Infectious Diseases.

[100] Carbone E, Terrazzano G, Meliv°n A (2000). Inhibition of Human NK Cell-Mediated Killing by CD1 Molecules. The Journal of Immunology.

[101] Campos-Martin Y, Gomez del Moral M, Gozalbo-Lopez B, Suela J, Marinez-Naves E (2004). Expression of Human CD1d Molecules Protects Target Cells from NK Cell-Mediated Cytolysis. The Journal of Immunology.

[102] Bozzano F, Costa P, Passalacqua G (2009). Functionally relevant decreases in activatory receptor expression on NK cells are associated with pulmonary tuberculosis in vivo and persist after successful treatment. Int Immunol.

[103] Vitale M, Della Chiesa M, Carlomagno S (2005). NK-dependent DC maturation is mediated by TNFalpha and IFNgamma released upon engagement of the NKp30 triggering receptor. Blood.

[104] Velez D, Wejse C, Stryjewski M (2010). Variants in toll-like receptors 2 and 9 influence susceptibility to pulmonary tuberculosis in Caucasians, African-Americans, and West Africans. Human Genetics.

[105] Texereau J, Chiche J-D, Taylor W, Choukroun G, Comba B, Mira J-P (2005). The Importance of Toll-Like Receptor 2 Polymorphisms in Severe Infections. Clinical Infectious Diseases.

[106] Xiong Y, Song C, Snyder GA, Sundberg EJ, Medvedev AE (2012). R753Q Polymorphism Inhibits Toll-like Receptor (TLR) 2 Tyrosine Phosphorylation, Dimerization with TLR6, and Recruitment of Myeloid Differentiation Primary Response Protein 88. Journal of Biological Chemistry.

[107] Sivori S, Carlomagno S, Moretta L, Moretta A (2006). Comparison of different CpG oligodeoxynucleotide classes for their capability to stimulate human NK cells. European Journal of Immunology.

[108] Sivori S, Falco M, Della Chiesa M (2004). CpG and double-stranded RNA trigger human NK cells by Toll-like receptors: induction of cytokine release and cytotoxicity against tumors and dendritic cells. Proc Natl Acad Sci U S A.

